# Case Report: Long-Term Survival of a Pediatric Patient With an Intra-Abdominal Undifferentiated Carcinoma of Unknown Primary

**DOI:** 10.3389/fonc.2021.590913

**Published:** 2021-05-10

**Authors:** Anja Stirnweiss, Hetal Dholaria, Joyce Oommen, Kathy Hardy, Gareth Jevon, Alex H. Beesley, Rishi S. Kotecha

**Affiliations:** ^1^ Telethon Kids Cancer Centre, Telethon Kids Institute, University of Western Australia, Perth, WA, Australia; ^2^ Department of Clinical Haematology, Oncology, Blood and Marrow Transplantation, Perth Children’s Hospital, Perth, WA, Australia; ^3^ Cyto Labs Pty. Ltd., Perth, WA, Australia; ^4^ Department of Anatomical Pathology, Perth Children’s Hospital, Perth, WA, Australia; ^5^ Curtin Medical School, Curtin University, Perth, WA, Australia

**Keywords:** carcinoma of unknown primary (CUP), FANCM2, SMARCD2, pediatric, case report

## Abstract

An 8-year and 10-month-old boy presented following 2 weeks of abdominal pain, vomiting, constipation, and rectal pain. A diffuse lower-abdominal mass was felt upon palpation, with radiological findings confirming the presence of a large, multilobulated intraperitoneal mass with mesenteric lymphadenopathy and hepatic metastatic disease. A biopsy of the mass revealed anatomical pathological findings consistent with a diagnosis of intra-abdominal undifferentiated carcinoma of unknown primary (CUP). The patient was treated with six cycles of carboplatin and gemcitabine prior to surgery. Following incomplete resection of the tumor, four further cycles were administered resulting in resolution of the pelvic mass, but progression in the right and left lobes of the liver. Therapy was accordingly adjusted, with administration of six cycles of ifosfamide and doxorubicin followed by 1 year of metronomic vinorelbine and cyclophosphamide maintenance therapy. The patient remains in remission 7 years from completion of therapy. Whole exome sequencing revealed missense mutations in the DNA-repair and chromatin-remodeling genes *FANCM* and *SMARCD2*, and a tumor-derived cell line revealed a complex karyotype suggesting chromosomal instability. CUP is an extremely rare diagnosis in the pediatric population, previously reported during adolescence. This report provides detailed characterization of CUP in a young child and in the absence of defined therapeutic guidelines for pediatric CUP, the successful treatment strategy described should be considered for similar cases.

## Introduction

Pediatric undifferentiated carcinoma is rare and associated with a poor prognosis (1, 2). The majority of histopathological subtypes fall within the Children’s Oncology Group (COG) definition of infrequent tumors (3), and classified under “other malignant epithelial neoplasms and melanomas” in the International Classification of Childhood Cancer (ICCC) subgroup XI of the Surveillance Epidemiology and End Results (SEER) database. Poor outcomes are reflected by the absence of scientific structures dedicated to identifying the underlying biology and optimal therapeutic strategies for pediatric carcinoma in general (4). In order to identify effective treatments and improve the outcome for rare tumors which have limited biological or clinical research representation, it is essential to undertake molecular characterization and report the clinical course of each individual case. We report a child with an undifferentiated large cell carcinoma of an unknown primary site (CUP) who remains in clinical and radiological remission 7 years following the end of multi-agent chemotherapy. Using tumor-normal whole exome sequencing we identified heterozygous missense mutations in the DNA-repair and chromatin remodeling genes *FANCM* and *SMARCD2*, suggesting that genetic instability may be a feature of this tumor.

## Case Presentation

**Figure 1:**
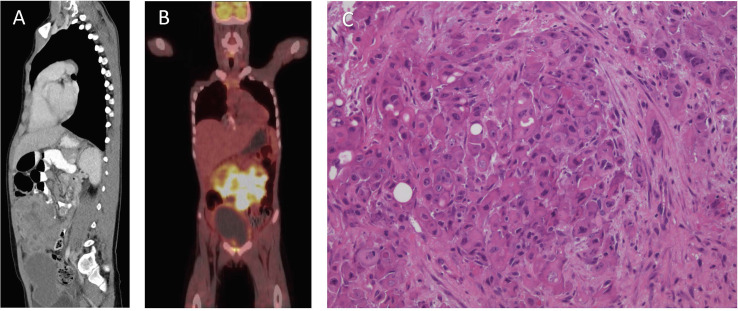
Clinical features of patient at diagnosis. **(A)** Computerized Tomography scan of the abdomen and pelvis. **(B)** Fluorodeoxyglucose–positron emission tomography scan. **(C)** Hematoxylin and eosin stain, showing nests of pleomorphic epithelioid cells with occasional vacuoles and luminal structures.

An 8-year and 10-month-old Caucasian boy presented to Princess Margaret Hospital for Children (Perth, Western Australia) following 2 weeks of abdominal pain, vomiting, constipation, and rectal pain. A diffuse lower-abdominal mass was felt upon palpation, and computerized tomography (CT) revealed a large anterior multi-lobulated intraperitoneal mass abutting the abdominal wall and extending from above the umbilicus to the colovesical pouch inferiorly, with multiple enlarged mesenteric nodes in the upper abdomen (**Figure 1A**). The mass demonstrated high-grade FDG-PET activity (**Figure 1B**), including the right lobe of the liver, suggestive of metastatic disease.

**Figure 2:**
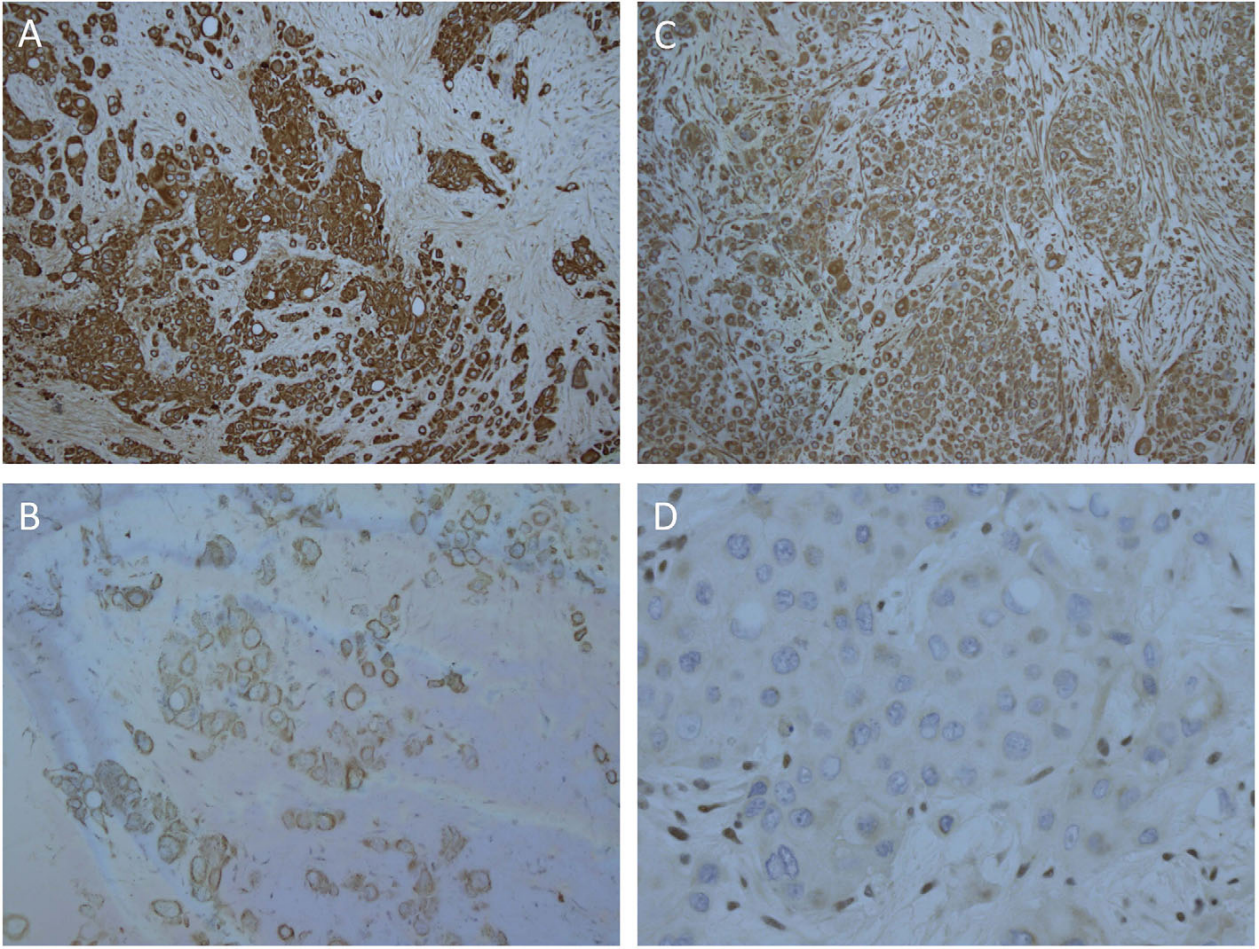
Strong, diffuse immunohistochemical staining of tumor cells. **(A)** CK AE1/AE3, **(B)** CK20, **(C)** Vimentin, **(D)** Loss of nuclear staining with INI1 and positive staining in the internal control stromal nuclei.

Biopsies of the abdominal mass were performed for diagnostic assessment. Histopathological assessment of three biopsy sites, revealed nests of pleomorphic, epithelioid cells with large nuclei surrounded by desmoplastic fibroconnective tissue with brisk mitotic activity. The tumor cells had a large amount of eosinophilic cytoplasm with well-defined cell borders, focal rhabdoid features, but no evidence of squamous differentiation. Some had large cytoplasmic vacuoles, and occasionally cells were arranged around a lumen (**Figure 1C**). Assessment of peritoneal fluid revealed malignant cells with the same cytological features. Cells from the abdominal mass stained strongly positive for CK AE1/AE3, CK20, and vimentin with loss of nuclear INI1 staining in most cells (**Figures 2A–D**). Immunohistochemical markers for squamous carcinoma (CK5/6, p63), pulmonary carcinoma (TTF1), NUT midline carcinoma (NUTM1), epithelioid sarcoma (CD117, CD34), melanoma (HMB45), and colonic and gastric adenocarcinoma (CEA, CDX2) were negative. INI1, BRD4-NUTM1, and EBV fluorescence *in situ* hybridization studies were normal. Coupled with ultrastructural findings on electron microscopy, the features were not diagnostic of any particular entity, but more in keeping with a poorly differentiated large cell CUP. Extensive investigations to determine the primary site of disease, including a magnetic resonance imaging scan of the bowel, CT scan of the sinuses, scrotal ultrasound, and upper and lower gastrointestinal endoscopies with biopsies, were inconclusive.

**Figure 3:**
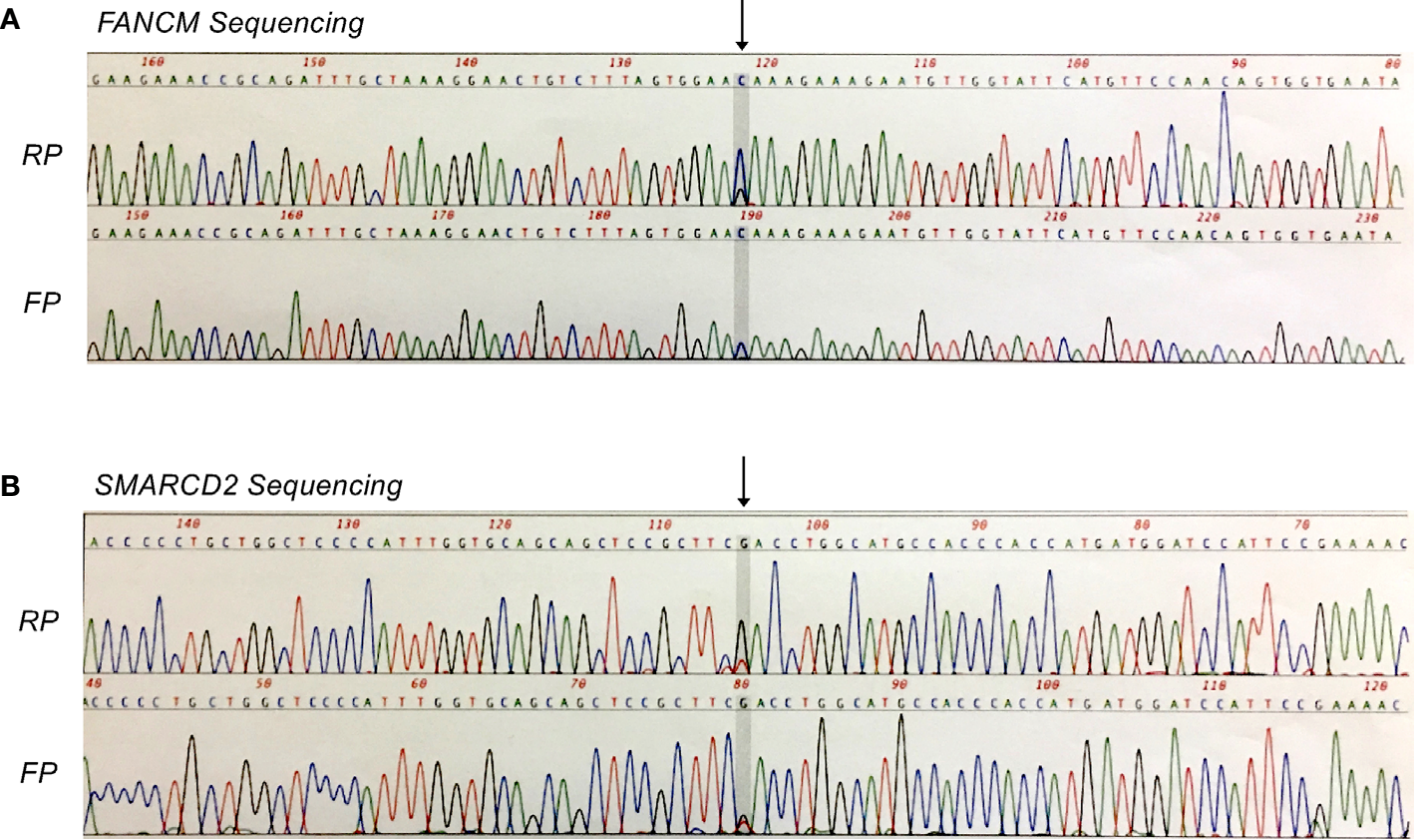
Sanger confirmation of missense mutations identified by whole exome sequencing analysis. **(A)** Heterozygous FANCM mutation (c.5857C>G, p.Gln1953Glu). **(B)** Heterozygous *SMARCD2* mutation (c.311G>T, p.Arg104Leu). FP, forward primer; RP, reverse primer.

To characterize genomic alterations, tumor and matched normal bone marrow DNA samples were analyzed *via* AmpliSeq Exome Sequencing on the Ion Proton platform (P1 Chip). Reads were mapped to hg19 using TMAP (Torrent Suite 4.2) and variants were called using Torrent Variant Caller and Ion Reporter 4.0. Baseline QC metrics for tumor and normal samples, respectively, were as follows: Total read count, 40.2 and 38.8 million; mean coverage depth, 115- and 110-fold; Ti/Tv ratio (SNPS), 2.475 and 2.472; total number of variants *vs.* hg19, 51,395 and 51,144 (SNVs, MNVs, Indels, CNVs). Subsequent tumor *vs.* normal comparison identified a total of 544 variants that were potentially unique to the tumor sample (516 SNVs, MNVs, and Indels; 28 CNVs). Confidence filtering based on p-value, allele read count and allele ratio reduced this list to 148 candidate variants for further consideration. Here, we focused on non-synonymous variations in protein coding-regions, being those most likely to be phenotypic (n = 34), and performed additional filtering based on variant allele frequency (VAF) and visual inspection of local sequence quality in IGV (Integrative Genomics Viewer, Broad Institute). This resulted in a shortlist of seven candidate missense variants (five SNVs, two Indels) for subsequent validation. Of these, two heterozygous missense mutations in the tumor-suppressor genes FANCM (c.5857C>G, p.Gln1953Glu) and SMARCD2 (c.311G>T, p.Arg104Leu) were confirmed by Sanger sequencing (**Figure 3**).

A cell line was grown from the patient’s tumor (PER-891) as previously described (5). DNA fingerprinting was used to confirm cell line identity (**Supplementary Table 1**). PER-891 demonstrated a complex karyotype [46,XY,t(12;22)(p12.2;p12), t(15;15)(p12;q11.2),+15] with numerous additional non-clonal changes indicating marked chromosomal instability. Drug-sensitivity profiling of the cell line revealed cytotoxicity to anthracyclines (daunorubicin IC_50_ 0.48 μM; mitoxantrone IC_50_ 0.27 μM), vinca alkaloids (vincristine IC_50_ 0.03 μM), topoisomerase I inhibitors (topotecan IC_50_ 0.73 μM), and the nucleoside analogue gemcitabine (IC_50_ 0.57 μM), which are drug classes conventionally used to treat CUP (5).

**Figure 4:**
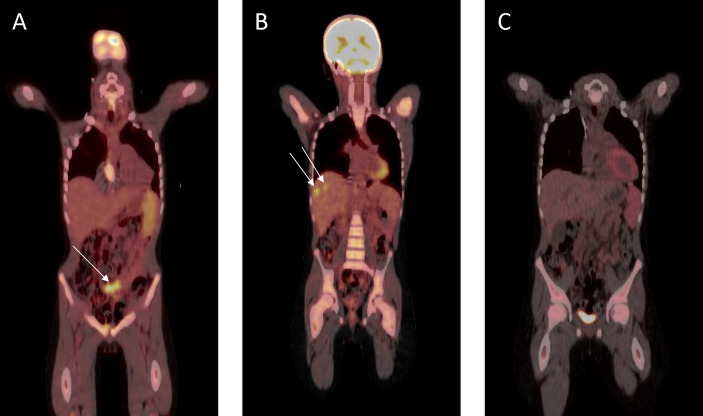
Fluorodeoxyglucose–positron emission tomography scan response assessment during therapy. **(A)** Very good partial response prior to surgical resection. **(B)** At hepatic progression (metastatic lesions to segments 7 and 8 shown). **(C)** Complete remission prior to metronomic maintenance therapy.

The patient was initially treated with four three-weekly cycles of carboplatin (AUC 5 mg/ml/min, Day 1) and gemcitabine (1 g/m^2^, Days 1 and 8). Repeat imaging showed a very good partial response (**Figure 4A**) and a further two cycles were administered prior to incomplete surgical resection of residual tumor, which included a pelvic mass attached to the bladder, an omental mass off the anterior abdominal mass, and small lymph nodes. Histopathological assessment of the pelvic mass revealed large areas of necrosis and minimal residual tumor surrounded by a fibrous tissue capsule. No tumor was detected on examination of three lymph nodes. However, the omental mass revealed viable tumor cells with morphological and immunohistochemical features consistent with the original diagnostic biopsy. Four further cycles of chemotherapy were administered following an uneventful post-operative period. Following completion of this therapy, abdominal CT and FDG-PET scans revealed a continued partial response, with resolution of the pelvic tumor, but progression in the right (segments 7 and 8) and left (segment 3) lobes of the liver (**Figure 4B**). Chemotherapy was subsequently adjusted to ifosfamide (3 g/m^2^, Days 1 to 3) and doxorubicin (37.5 mg/m^2^, Days 1 and 2), repeated every 4 weeks. FDG-PET revealed a complete metabolic response following two cycles of therapy, and a further four cycles were administered, resulting in complete radiological remission (**Figure 4C**). The patient was subsequently treated with metronomic maintenance therapy for 1 year, comprising of four weekly cycles of vinorelbine (25 mg/m^2^, Days 1, 8, and 15) and oral cyclophosphamide (25 mg/m^2^, Days 1 to 28). He remains in continued complete clinical and radiological remission 7 years following the completion of therapy.

## Discussion

Carcinoma of unknown primary origin (CUP) describes a heterogeneous group of cancers determined to be metastatic at diagnosis but for which a primary tumor cannot be identified based on a full standardized diagnostic workup. Clinical features of CUP include a short history with symptoms and signs associated with metastatic sites, early dissemination in the absence of primary tumor and an aggressive clinical course (6). In adults they account for 2–5% of all malignancies, however they are extremely rare in children (7). Lymphoma, germ cell tumors, neuroblastoma, and rhabdomyosarcoma represent the most commonly occurring differential diagnoses that may be considered at first presentation in children. As CUP is a diagnosis of exclusion, staging investigations and pathological assessment for other rare pediatric carcinomas such as hepatocellular, renal cell, nasopharyngeal, thyroid, colorectal, skin, adrenocortical, and NUT-midline carcinoma should be performed prior to a diagnosis being made. To achieve optimal survival and quality of life, children with such rare tumors should be managed and treated in a tertiary pediatric cancer center so that they can benefit from a multidisciplinary team approach, with access to healthcare professionals that have expertise in treating and caring for children with cancer.

In the present case, an 8-year and 10-month-old boy with multiple abdominal masses was diagnosed with poorly differentiated CUP. With a median survival rate of 4 months and a 5-year overall survival rate of 10%, the prognosis for patients with CUP is historically poor. However, the National Cancer Institute’s PDQ cancer information summary regarding CUP states that a small subset of patients with poorly differentiated CUP are potentially curable (8). Three characteristics reported to have an association with good prognosis in this disease were present in our index case, namely: (i) age younger than 50 years; (ii) a midline tumor distribution; and (iii) clinical evidence for rapid tumor growth. Platinum, gemcitabine, and taxane-based combinations comprise the standard chemotherapeutic agents which are currently used to treat CUP (9–14). Irinotecan and vinorelbine have also been used as part of combination therapy (13–15). In the case presented here, the initial response to carboplatin and gemcitabine was only partial. Given that the patient achieved durable remission after changing to ifosfamide and doxorubicin followed by metronomic maintenance therapy with vinorelbine and cyclophosphamide, this treatment regimen might be beneficial for patients with non-responsive, poorly differentiated CUP lesions.

Genetic characterization of the tumor with whole exome sequencing revealed two heterozygous mutations in the tumor-suppressor genes *FANCM* and *SMARCD2*. *FANCM* is a member of the Fanconi Anemia (FA) gene family and plays an important role in the repair of DNA interstrand cross-links (16). Heterozygous mutations in various FA genes have been shown to be associated with an increased cancer risk, with Kiiski and colleagues reporting a *FANCM* mutation that was associated with a two-fold increase in risk of breast cancer, especially triple-negative breast cancer, in the Finnish population (17). SMARCD2 is a component of the SWI/SNF chromatin-remodeling complex, which mobilizes nucleosomes to facilitate activation, as well as repression, of gene transcription (18, 19). Moreover, it has been shown that mutations in *SMARCD2* correlate with the level of DNA damage in certain populations (20). We used strict filtering criteria to identify true variants associated with the tumor sample. Whilst the mutations in FANCM and SMARCD2 were the only variants to be independently validated, it is likely that there may be additional genomic variations in this tumor below the level of detection provided by our analysis, potentially as the result of tumor cellular heterogeneity or local genomic region complexity. While further studies are warranted to understand the clinical implications of these mutations in undifferentiated CUP, the heterogeneity of this patient group suggests that those with CUP may represent ideal candidates for personalized therapy approaches informed by genetic sequencing (21). The feasibility of personalized treatment has recently been demonstrated in a retrospective analysis of 303 adult CUP samples, which identified several key genomic aberrations that could be matched to targeted therapy in approximately 32% of cases (7). This approach is currently being investigated in the ongoing CUPISCO randomized phase 2 trial for adults with newly diagnosed CUP, which is comparing the efficacy and safety of targeted therapy or immunotherapy, guided by comprehensive genomic profiling, versus standard platinum-based chemotherapy (NCT03498521). Although disease rarity precludes the conduct of clinical trials specifically for children with CUP, the recent development of national and/or institutional platforms have facilitated delivery of personalized therapeutic approaches on an individual basis (NCT03155620; NCT03336931) (22, 23).

Collaborative research efforts towards understanding tumor biology and therapeutic targets in cancers of unknown primary site is an unmet area of need. Children represent less than 1% of all solid cancers of unknown primary site (2). Knowledge of clinical presentation, management, and long-term outcome is based on limited pediatric data or extrapolation from adult studies. The gap in clinical and scientific knowledge for such rare pediatric tumors has been recognized with formation of specialized task forces, including the European Cooperative Study Group for Pediatric Rare Tumors (EXPeRT) and the Children’s Oncology Group Rare Tumors Committee, that are best poised to conduct collaborative clinical and biological research to improve the knowledge and outcome of children with CUP.

## Data Availability Statement

The original contributions presented in the study are included in the article/**Supplementary Material**. Further inquiries can be directed to the corresponding author.

## Ethics Statement

The studies involving human participants were reviewed and approved by the Princess Margaret Hospital Human Research Ethics Committee. Written informed consent to participate in this study was provided by the participants’ legal guardian/next of kin. Written informed consent was obtained from the minor(s)’ legal guardian/next of kin for the publication of any potentially identifiable images or data included in this article.

## Author Contributions

AS, AB, and RK conceived and designed the study. HD, GJ, and RK provided study materials and patient information. KH performed and interpreted the cytogenetic analysis. AS, JO, and AB conducted, analyzed, and interpreted data for exome sequencing. AS, HD, AB, and RK wrote the manuscript. All authors contributed to the article and approved the submitted version.

## Funding

RK is supported by a Fellowship from the National Health and Medical Research Council of Australia (NHMRC APP1142627). This study was supported by the Children’s Leukaemia and Cancer Research Foundation (CLCRF), Perth, Australia.

## Conflict of Interest

Author KH was employed by the company Cyto Labs Pty. Ltd.

The remaining authors declare that the research was conducted in the absence of any commercial or financial relationships that could be construed as a potential conflict of interest.
